# Nucleotide sequence variants, gene expression and serum profile of immune and antioxidant markers associated with brucellosis resistance/susceptibility in Shami goat

**DOI:** 10.1186/s13620-025-00285-4

**Published:** 2025-01-18

**Authors:** Ahmed A. Elsayed, Ahmed M. Sallam

**Affiliations:** 1 Animal and Poultry Production Division, Animal Health Department, Desert Research Center, Cairo, Egypt; 2 Animal and Poultry Production Division, Department of Animal and Poultry Breeding, Desert Research Center, Cairo, Egypt

**Keywords:** Genetic markers, Antioxidants, Immunity, Single nucleotide polymorphisms, Gene expression, Brucella

## Abstract

Brucellosis is a highly contagious zoonotic bacterial disease. It has considerable negative consequences on the animal production industry worldwide. The objective of this study was to investigate the genetic and molecular variations in Shami goat susceptible to *Brucella* infection. Blood samples were collected from fifty mature Shami goats (30 *Brucella*-infected does and 20 non-infection). DNA was extracted and selected parts the immunity; *solute carrier family 11 member 1 (SLC11A1)*,* toll-like receptor 1 (TLR1)*,* toll-like receptor 9 (TLR9)*,* SP110 nuclear body protein (SP110)*,* the adenosine A3 receptor (ADORA3)*,* caspase activating recruitment domain 15 (CARD15)* and *interferon regulatory factor 3 (IRF3)*,* antioxidant glutathione peroxidase 1 (GPX1)*,* nitric oxide synthase (NOS)*,* NAD(P)H dehydrogenase [quinone] 1 (NQO1)* and *transcription factor NF-E2-related factor 2 (Nrf2) and erythritol related transketolase (TKT)*,* ribose 5-phosphate isomerase (RPIA) and Adenosine monophosphate deaminase (AMPD)* genes were sequenced. Likewise, the levels of gene expressions were investigated. The results identified polymorphic variants between healthy and infected does. Levels of gene expression of *SLC11A1*,* TLR1*,* TLR9*,* SP110*,* ADORA3*,* CARD15*,* IRF3*,* HMOX1*,* TKT*,* RPIA* and *AMPD* were significantly (*P* < 0.05) up regulated in the infected compared to the non-infected ones. On the other hand, *GPX1*, *NOS*, *NQO1* and *Nrf2* genes were significantly (*P* < 0.05) downregulated in the infected compared to the non-infected does. The results of serum profile indicated that there is a significant (*P* < 0.05) increase in the activities of AST, ALT, GGT, LDH, ALP as well as serum level of globulin, triglycerides, cholesterol, MDA, NO, IL-1β, TNF-α, IgM, IgG, haptoglobin and amyloid A. On the other hand, there were significant reductions in the glucose, total protein albumin, urea, calcium, inorganic phosphorus, sodium, copper, zinc, iron, TAC, GSH, SOD, GPx, IL-10 and fibrinogen in the infected compared to the non-infected does. Our results provide valuable information about the serum profile variations and putative genetic markers for *Brucella* infection in goats. This could be utilized in controlling goat brucellosis through selective breeding of natural resistant animals.

**Supplementary Information:**

The online version contains supplementary material available at 10.1186/s13620-025-00285-4.

## Introduction

Goat is an important part of animal production industry, particularly in arid regions [1]. The total goat population in Egypt was estimated as about 3.4 million heads [2]. While goats are not prominent for milk when compared to buffaloes and cattle, there is an increasing demand for goat dairy products, especially cheese [3]. Shami goats, also referred to as Damascus goats, are known with their high productive and reproductive capabilities [4, 5]. Moreover, they are adapted to live in a wide range of environmental temperatures, especially the arid regions [6]. It has a genetic potential as a dual-purpose breed (i.e. meat and milk) [7], therefore, it has been involved in genetic improvement programs of local breeds in many countries worldwide [8].

Brucellosis is a worldwide highly contagious bacterial infectious disease [9]. It has been reported by the World Health Organization as a neglected zoonosis, which means it does not receive sufficient attention and resources despite its global impact [10]. *Brucella* is endemic in Egypt and regularly widespread in humans and livestock across the country [11, 12]. In goats, *Brucella melitensis* is the most common causative agent of the disease. The bacterium is gram-negative and facultative intracellular, which means it can survive and reproduce inside the host cells [13]. The disease can transmit from the infected animals to healthy animals and humans through direct contact, contaminated materials and animal products [14]. The transmission can occur through different routes, including the conjunctiva, digestive and respiratory systems [15]. Once the *Brucella* enters the body, it can travel through the lymphatic and blood circulation systems to the regional lymph nodes and then spread to various organs including, the spleen, liver, bone marrow, central nervous system and reproductive organs [16].

Brucellosis causes significant economic losses such as abortion, mastitis, reduced milk production and reproductive problems [9, 12, 17–19]. While symptoms during the chronic phase of the disease are typically not pathognomonic [20]. Many infected animals may be possible carriers of the disease due to the chronic presence of the *Brucella* pathogen in their supramammary lymphatic nodes and mammary glands [21], resulting in continued secretion of the organism in their body fluids [9]. The absence of an effective cure and challenges with vaccination is primarily due to the low sensitivity and specificity of the serological diagnostic tests [22]. Accordingly, identification of genetic markers for *Brucella* may deepen our understanding of the disease and developing new tools of detection [23].

The advanced molecular genetic techniques could help as adjunct to control the disease by improving animal health [24]. Several genetic markers, mostly single nucleotide polymorphisms (SNPs), have been successfully pinpointed for assessing the disease susceptibility in livestock [25–30]. This suggests the existence of variations among animals in their susceptibility to the disease, which could be attributed to genetic variations [31]. The idea of a selection criterion in genomic techniques to promote disease resistance is shifted from phenotypically expressed illness state to allele one at the DNA level, which is called marker-assisted selection (MAS). MAS offers an excellent opportunity for selecting genetically resistant animals since it increases selection accuracy and allows for selection without subjecting animals to disease challenges [32].

Polymorphic variations and changes in the expression levels of immunity- related genes responding to *Brucella* in goats have been well documented [33]. In goats [30] as well as in other species [34–39], polymorphic variations and changes in the expression levels of immunity and antioxidants-related genes responding to Brucella have been well documented. Nonetheless, there is limited information available regarding the immunological and antioxidant changes, SNPs and gene expression profiles linked to goat brucellosis. Therefore, the objective of this study was to investigate potential genetic polymorphisms and differentially expressed genes, along with immunological and antioxidant alterations associated with *Brucella* infection in goats.

## Materials and methods

**Herd management**. All animal procedures included in the current study were approved by the animal breeding committee and Animal Health and Poultry Department (Approval No.9, March 2020) at the Desert Research Center (DRC) in Egypt. All methods were performed in accordance with the relevant ARRIVE guidelines and regulations (https://arriveguidelines.org/). This study involved a total of 50 adult Shami goats with an average of 4.9 ± 0.7 years and average body weight of 49.16 ± 6.5 kg. Water was always accessible to the does, and they were kept in semi-open shaded pens with 750 g of concentrate feed mixture (CFM) + 750 g of alfalfa hay/head/day for feeding. Table 1 displays the ingredients of the basal diet. When available, the natural pasture which consisted of grass, berseem, darawa, and green herbage was fed. The does that had normal lambing and normal postpartum stage (i.e., normal feed intake, body temperature, no uterine discharge and normal udder) were considered as *Brucella* non-infected (*n* = 20). The does that suffered from abortion at the last third period of pregnancy with retained placenta was considered *Brucella* infected (*n* = 30). Comprehensive information regarding animal age, movement, health status, client complaints, herd size, disease history and reproductive problems, such as abnormal uterine discharge and abortion, was recorded. All animals received the same diets and were managed under the same management system.

**Table 1:** Composition of the concentrate feed mixture (CFM)

Ingredients	Quantity
Corn	530 kg
Wheat bran	240 kg
Soya bean	230 kg
Sodium chloride	5 kg
Calcium carbonate	10 kg
Premix	1 kg
Netro-Nill	0.5 kg
Fylax	0.5 kg

### Blood sampling

Blood samples were collected from each doe in the study at 8 O’clock morning via jugular vein into plain tubes without anticoagulants and tubes containing EDTA. The total DNA, RNA and complete blood count (CBC) were extracted immediately from the whole blood of each sample. To detect the *Brucella*, serum samples were initially screened using Rose Bengal Plate test (RBPT) [40], followed by a further confirmed using Serum Tube Agglutination test (STAT) [41]. The RBPT was performed according to the laboratory Standard Operating Procedures (SOP) based on the World Organization of Animal Health manual (World Organisation for Animal Health (OIE), 2019) [42]. Briefly, equal volumes (30 µL) of standardized *B. melitensis* antigen and test serum were thoroughly mixed for 4 min and the appearance of agglutination recorded as a positive result. Positive samples were categorized based on the degree of agglutination, which ranged from weakly positive (+) to strongly positive (++++). Samples that did not show agglutination was within 4 min were considered negative (−). Using STAT, significant titers were defined as those with a value of ≥ 1/80 [43]. Seropositivity was only confirmed when serum samples were reacted positively in both RBPT and STAT tests. Samples that were negative results to either RBPT or STAT were classified seronegative. Finally, 20 healthy does who gave normal birth and were tested negative in both RBPT and STAT tests were diagnosed *Brucella* non-infected. Thirty does were tested positive to both RBPT and STAT and showed abortion at the last third part of pregnancy were considered *Brucella melitensis* infected.

### DNA extraction and polymerase chain reaction (PCR)

Genomic DNA was extracted from whole blood using Gene JET Whole Blood Genomic DNA Extraction Kit, following the manufacturer instructions (Thermo Scientific, Lithuania). Subsequently, the DNA was evaluated for quality, purity and concentration using a Nanodrop spectrophotometer (≥ 50 ng/µL).

PCR was conducted to amplify the coding regions of genes related to immunity *(SLC11A1*,* TLR1*,* TLR9*,* SP110*,* ADORA3*,* CARD15* and *IRF3)*, antioxidant (*GPX1*, *NOS*,* HMOX1*, *NQO1* and *Nrf2*) and erythritol (*TKT*,* RPIA* and *AMPD*). The primer sequences (Supplementary Table 1) were designed based on the *Capra hircus* genome assembly (https://www.ncbi.nlm.nih.gov/nuccore/?term=Capra+hircus+genome), available on the National Center for Biotechnology Information (NCBI). The thermal cycler PCR reaction mixture was performed in a final volume of 100 µL, combrising 5 µL DNA, 43 µL distilled water (H_2_O), 50 µL PCR master mix (Jena Bioscience, Germany), and 1 µL of each primer. The PCR reaction consisted of an initial denaturation step at 95 °C for 6 min, followed by 35 cycles of denaturation at 95 °C for 45 s, annealing temperature for 1 min as specified in Table S2, and extension at 72 °C for 45 s and a final extension at 72 °C for 10 min. Subsequently, DNA fragment patterns of the PCR product were detected by agarose gel electrophoresis.

### DNA sequencing and polymorphism detection

The PCR products were purified using PCR purification kit following the manufacturer instructions (Jena Bioscience, Germany). The desired DNA fragment was taken off of an agarose gel, put in a microcentrifuge tube, dissolved in binding buffer, and then put on the column. The binding buffer’s chaotropic agent facilitates DNA binding to the silica membrane in the column, denatures proteins, and dissolves agarose. All impurities were eliminated using a quick wash procedure. After that, the elution buffer was used to elute the purified DNA from the column. Then, the quantification and purification of the PCR products were performed using a Nanodrop spectrophotometer. Subsequently, the PCR products were sequenced in both the forward and reverse directions using ABI 3730XL DNA sequencer (Applied Biosystem, USA). The resulting DNA sequences were examined using Chromas 1.45 and BLAST 2.0 software [44].

Instead of using radioactive labels to identify the dideoxynucleotides (ddNTPs), four distinct fluorescent labels were used for the sequencing reaction. During the electrophoresis, two argon lasers were used to activate these fluorophores at 488 and 514 nm, respectively, when the corresponding bands passed the lasers. The particular emissions were found, and information was gathered for examination. Polymorphisms were detected by aligning the obtained sequences with the corresponding gene from the *Capra hircus* reference genome using the MEGA4 software [45].

### Total RNA extraction and quantitative real time PCR

For each whole blood sample, the total RNA was extracted using Trizol reagent following the manufacturer’s protocol (RNeasy Mini Ki, Catalogue no.74104). We then assessed the quality and quantity of the extracted RNA using the NanoDrop^®^ND-1000 Spectrophotometer. Subsequently, we synthesized the cDNA of each sample in accordance with the manufacture protocol (Thermo Fisher, Catalog no, EP0441).

To determine the relative mRNA levels of the target genes, RT-PCR was conducted using SYBR Green PCR Master Mix (Quantitect SYBR green PCR kit, Catalog no, 204141). The primer sequences were designed based on the reference genome of *Capra hircus* presented in Supplementary Table S2. To ensure accurate normalization, the *ß. actin* housekeeping gene was used as a constitutive control.

For each sample, the 25 µl total reaction volume consisted of a mixture of 3 µl of total RNA, 4 µl of 5x Trans Amp buffer, 0.25 µl reverse transcriptase, 0.25 µl of each primer, 12.5 µl 2x Quantitect SYBR green PCR master mix, and 4.75 µl RNase free water. The thermal cycler PCR was used to amplify the target sequence following this program: Initial reverse transcription at 50 °C for 30 min, primary denaturation at 94 °C for 10 min followed by 40 cycles of 94 °C for 15 s, annealing at temperatures specified in Supplementary Table S2, and extension at 72 °C for 30 s. After the amplification, a melting curve analysis was conducted to confirm the specificity of each PCR product. The relative expression of each gene for each sample in comparison with *ß. actin* gene was determined and calculated using the 2^−ΔΔCt^ method [46].

### Biochemical analysis

Serum biochemical analyses was conducted using commercial test kits in accordance with standard protocols provided by the respective supplier. The following kits were used to measure the serum levels concentration of various components:

1. Total protein, albumin, glucose, cholesterol and creatinine were quantified using kits from Gamma Trade Company, Egypt. Calcium, phosphorus, and magnesium were determined using kits from Bio-Diagnostic in Egypt.
2. Sodium, potassium, chloride, triglyceride, urea, AST (aspartate aminotransferase), ALT (alanine aminotransferase), ALP (alkaline phosphatase), LDH (lactate dehydrogenase) and GGT (gamma glutamyl transferase) levels were measured with kits from Spectrum Company in Egypt using a selective chemistry analyzer (Apple 302, USA). Globulin was calculated by subtracting albumin values from total serum protein concentration.
3. Additional parameters, such as malondialdehyde (MDA), glutathione peroxidase (GPx), nitric oxide (NO), super oxide dismutase (SOD), total antioxidant capacity (TAC), glutathione reduced (GSH), IL1-β, IL-6 and IL-10 and TNF-α were assessed using specific kits from various suppliers. Immunoglobulin G (IgG) was measured using a kit from Cell Sciences company and Immunoglobulin M (IgM) using a kit from Genemed Synthesis.
4. Serum amyliod A (SAA), plasma fibrinogen (Fb) and haptoglobin (Hp) concentrations were determined using ELISA kits from IBL International Crop (Canada) and Eagle Biosciences (Columbia), respectively. Levels of Cu, Zn and Fe were assessed using kits from Sigma-Aldrich Co., Abnova Co. and Abcam Co., respectively.

### Statistical analysis

Null hypothesis (Ho): There is no association between SNPs, gene expression and serum profile of immune and antioxidant markers and brucellosis susceptibility in Shami goat. Alternative hypothesis (HA): There is an association between SNPs, gene expression and serum profile of immune and antioxidant markers and brucellosis susceptibility in Shami goat. Statistical analysis was conducted using the student’s t-test implemented in the SPSS version 20 software (Chicago, USA). Descriptive statistics were performed for all parameters at statistically significant level of P ˂ 0.05.

## Results

### Brucella incidence

According to the RBPT and STAT tests, 60% of Shami goats examined in the studied region tested positive to *Brucella* while 40% of the does that were negative to *Brucella*.

### DNA sequencing of the investigated genes

Polymorphic variations were identified in the genes related to immunity, antioxidant and erythritol in the DNA sequences of both brucellosis infected and non-infected does. These polymorphisms are detailed in Supplementary Table S3. The investigated genes including *SLC11A1* (523-bp), *TLR1* (471-bp), *TLR9* (460-bp), *SP110* (537-bp), *ADORA3* (521-bp), *CARD15* (394-bp), *IRF3* (468-bp), *GPX1* (420-bp), *NOS* (332-bp), *NQO1* (466-bp), *Nrf2* (480-bp), *TKT* (414-bp), *RPIA* (338-bp), and *AMPD* (382-bp) genes showed nucleotide sequence variations, which are polymorphic SNPs that may be associated with brucellosis susceptibility in the does under investigation. In contrast, the DNA sequencing of *HMOX1* (867-bp) exhibited a consistent, non-variable pattern (i.e., monomorphic). The variants identified are all located within exonic region of studied genes; resulting in coding mutations between healthy and brucella affected does (Table S3).

**Fig. 1:**
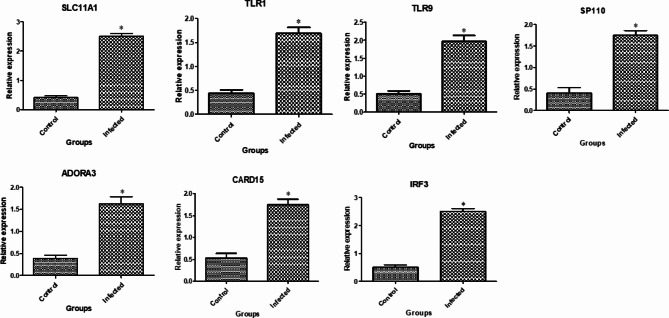
Gene expression profiles of the immune-related markers in the healthy and *Brucella* infected goats

**Fig. 2:**
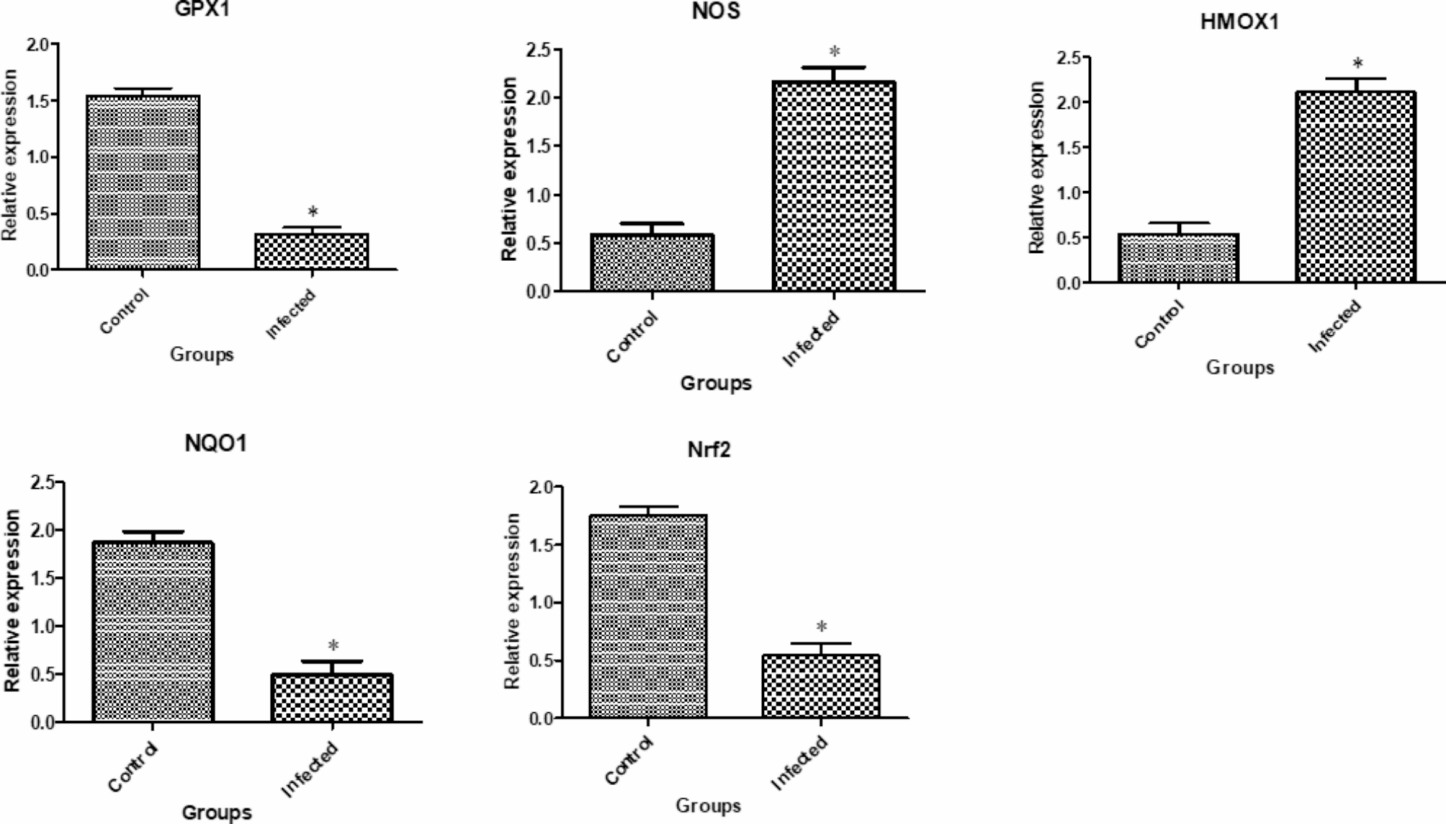
Gene expression profiles of the antioxidant-related markers in the healthy and *Brucella* infected goats

**Fig. 3:**
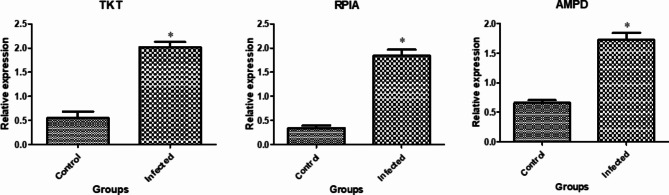
Gene expression profiles of the erythritol-related markers in the healthy and *Brucella* infected goats

**Patterns of gene expression.** Figs. 1, 2 and 3 display the gene expression profiles of markers related to immune, antioxidant and erythritol. In brucellosis infection in does, the *SLC11A1*,* TLR1*,* TLR9*,* SP110*,* ADORA3*,* CARD15*,* IRF3*,* HMOX1 TKT*,* RPIA* and *AMPD* genes exhibited a significant (P-value < 0.05) upregulation compared to non-infected does. Conversely, the *GPX1*, *NOS*, *NQO1* and *Nrf2* genes were significantly downregulated.

### Biochemical profile

In brucellosis infected does, a significant (P-value < 0.05) decrease was observed in both RBCs and Hb concentration while there were no significant changes observed in HCT, MCV, MCH and MCHC levels compared to non-infected does. Additionally, the infected does revealed a highly significant increase (P-value < 0.05) in the counts of leucocytes, neutrophils and monocytes with no significance changes in basophils, eosinophils and lymphocytes when compared to non-infected does (Table 2). In terms of biochemical findings, the study revealed a significant (P-value < 0.05) increase in the serum activity of AST, ALT, GGT, LDH, ALP and serum levels of globulin, triglycerides, cholesterol. Simultaneously, there was a significant decrease in the serum values of glucose, total protein albumin, urea, calcium, inorganic phosphorus, sodium, copper, zinc and iron in brucella infected when compared with non-infected does (Table 3).

**Table 2:** Hematological changes in the healthy and *Brucella* infected does

parameters	Healthy	Brucella infected	*P* values
**WBC(×10** ^**9**^ **/L)**	7.9 ± 1.6	11.7 ± 0.7*	0.006
**RBC (× 10** ^**12**^ **/L)**	11.8 ± 1.4	9.2 ± 1.1*	0.02
**Hb (g/dl)**	12.3 ± 0.8	9.8 ± 1*	0.008
**PCV%**	33.5 ± 1.2	30.5 ± 2.8	0. 1
**MCV (fL)**	30.7 ± 3.2	31.7 ± 3.4	0.68
**MCH (pg)**	10.4 ± 0.7	10.3 ± 1.2	0.82
**MCHC (g/dl)**	34.1 ± 1.5	32.3 ± 0.9	0.09
**lymphocyte (× 10** ^**9**^ **/L)**	2.6 ± 0.5	2.8 ± 0.3	0.4
**monocyte (× 10** ^**9**^ **/L)**	0.09 ± 0.02	0.25 ± 0.06*	0.01
**neutrophil (× 10** ^**9**^ **/L)**	4.7 ± 1.2	8.4 ± 0.9*	0.003
**Eosinophils (× 10** ^**9**^ **/L)**	0.09 ± 0.005	0.1 ± 0.0.01	0.3

* Significant at P-value < 0.05

**Table 3:** Some biochemical parameters in the healthy and *Brucella* infected does

Parameters	healthy (*n* = 20)	*Brucella* infected (*n* = 30)	*P*-value
**Glucose (mg/dl)**	84.6 ± 4	60 ± 3.2*	0.009
**Cholesterol (mg/dl)**	77.6 ± 8.6	116 ± 2.6*	0.01
**Triglyceride (mg/dl)**	53.6 ± 4.2	79.3 ± 4.4*	0.01
**TP (g/dl)**	5.8 ± 0.3	4.5 ± 0.2	0.02
**Albumen (g/dl)**	3.3 ± 0.2	2.3 ± 0.1*	0.01
**Globulin (g/dl)**	1.5 ± 0.18	3.2 ± 0.3*	0.01
**Urea (mg/dl)**	55.6 ± 4.1	33.3 ± 3.7*	0.01
**Creatinine (mg/dl)**	1.1 ± 0.1	1.4 ± 0.05	0.1
**AST (U/L)**	54 ± 5.2	82.6 ± 6.7*	0.02
**ALT (U/L)**	27.6 ± 2	65 ± 11.3*	0.02
**GGT (U/L)**	18.6 ± 4.1	31.9 ± 1.5*	0.03
**ALP (U/L)**	48.6 ± 3.1	143.6 ± 31.8	0.04
**LDH (U/L)**	222.8 ± 1	262.6 ± 6.3*	0.009
**Ca (mg/dl)**	6.7 ± 0.2	5.2 ± 0.2*	0.01
**Inorganic P (mg/dl)**	5.3 ± 0.4	3.6 ± 0.4*	0.04
**Mg (mg/dl)**	2.6 ± 0.1	2.8 ± 0.2	0.4
**Na (mmol/l)**	144.3 ± 2.9	124.6 ± 5.3*	0.03
**K (mmol/l)**	5.2 ± 0.3	5.3 ± 0.4	0.6
**Cu (µg/dL)**	88.34 ± 1.2	64.3 ± 1*	0.001
**Zn (µM)**	86.8 ± 1.9	63 ± 1.3*	0.001
**Fe (nmol)**	273.6 ± 3.5	166.3 ± 5.5*	0.001

* Significant at P-value < 0.05

Regarding the oxidative stress biomarkers, MDA and NO exhibited a significant increase while TAC, GSH, SOD, and GPx showed a significant decrease in brucella infected compared to non-infected does (Table 4). Serum values of IL-1β, TNF-α, IgM and IgG showed a significant (P-value < 0.05) increase while IL-10 showed a significant decrease (P-value = 0.027) in *Brucella* infected when compared with non-infected does. Additionally, the serum values of haptoglobin and amyloid A exhibited a significant (P-value < 0.05) increase while the fibrinogen displayed a significant decreased (P-value = 0.027) in *Brucella* infected compared to non-infected does (Table 5).

**Table 4:** Serum oxidative stress markers (mean ± SE) in the healthy and *Brucella* infected does

Parameter	healthy (*n* = 20)	*Brucella* infected (*n* = 30)	*P* values
**GSH (mg/dl)**	44 ± 3.2	25.6 ± 4*	0.02
**GPx (U/mL)**	56 ± 4.7	33.3 ± 3.5*	0.01
**MDA (nmol / mL)**	5.8 ± 0.5	9.4 ± 0.6*	0.01
**NO (µmol / L)**	5.7 ± 0.5	10.1 ± 0.8*	0.01
**SOD (U/ml)**	66.3 ± 3.4	42.6 ± 4.2*	0.01
**TAC (mM /L)**	46 ± 3.4	25.6 ± 4.4*	0.02

* Significant at P-value < 0.05

**Table 5:** Mean values and standard errors of cytokines and APP levels in the healthy and *Brucella* infected does

Parameter	healthy (*n* = 20)	*Brucella* infected (*n* = 30)	*P*-value
**IL 1 β (pg/ml)**	59.6 ± 3.7	96.6 ± 7.2*	0.01
**IL 6 (pg/ml)**	15.8 ± 2.2	77.3 ± 5*	0.001
**IL 10 (pg/ml)**	74.6 ± 4.3	49 ± 5.2*	0.02
**TNFα (pg/mL)**	24 ± 3.2	76 ± 5.5*	0.001
**IgG (ng/ml)**	29 ± 2	48 ± 6.3*	0.001
**IgM (ug/ml)**	5.3 ± 0.3	7.7 ± 0.3*	0.007
**Hp (ng/ml)**	52.3 ± 3.3	90.3 ± 2.4*	0.001
**SAA (mg/l)**	4.2 ± 0.5	7.3 ± 0.3*	0.009
**Fb (g/L)**	7.7 ± 0.1	9.6 ± 0.3*	0.007

* Significant at P-value < 0.05

## Discussion

The high prevalence ratio (60%) of brucellosis revealed in this study may be due to the large-scale animal grazing and mixed breeding practices, which create the optimum conditions for infection transmission within the herd. Furthermore, lack of animal tracking, unreported outbreaks, insufficient vaccination coverage, and ineffective management practices in the studied area may also play a role in the high spread of the disease, as suggested by previous studies [12, 18, 47]. Similar findings were also previously documented [48], although lower estimates (36%) were observed by Mahboub H. et al. [49] in the Nile Delta of Egypt.

In a previous report, we identified potential SNPs, candidate genes related to immunity and genomic regions that underly genetic variations between goats in brucellosis infection [30]. In the current study, sequencing the coding regions of the immunity-relate genes revealed novel SNPs that distinguish between brucellosis resistant and infected does. So far, this study marks the first instance where these SNPs were identified in the genes under investigation as potential contributors to brucellosis infection in goats. Notably, previous studies identified polymorphisms in the *SLC11A1* [50], *IRF3* [51], *TLR5* [29] and *PTPRT* [52] genes significantly associated with *Brucella* infection in goats using different genetic approaches. Additionally, several variants in cytokines (such as, *IFNG*) and innate immunity (such as *SLC11A1*, *TLR1*, *TLR4*, and *TLR9*) related genes were associated with in humans [53] goats [36] and cattle [54, 55]. It is worth noting that previous research has indicated that polymorphisms in the coding region of the *SLC11A1* gene could stimulate the resistance or susceptibility to bovine brucellosis [56] in vitro, not only in bovines like the Indian Zebu (*Bos Indicus*) but also crossbred (*Bos Indicus* × *Bos Taurus*) cattle [57] and buffalo [58].

Mutations are the main source of selection and adaptability [59]. Exonic mutations were found in all immunological, antioxidant and erythritol indicators under study. This may have altered the coding DNA sequences in brucella infected does compared to healthy ones [59]. Genetic variation caused by non-synonymous SNPs modifies the encoded amino acid at the mutant site, which can lead to structural and functional changes in the protein mutation [60].

Our findings concerning the expression levels of the investigated genes revealed that the genes related to inflammation and erythritol exhibited higher expression in infected does compared to non-infected. However, a contrasting pattern was observed for antioxidant genes except for *HMOX1*. The conservation behavior in the *HMOX1* gene could be explained by the close relatedness of ruminant species [61] and sequencing a conserved region of the gene [62].

It is worth noting that no information was available to compare with our results. Moreover, our results mark the first instance of combining SNP markers and gene expression approaches to identify polymorphisms linked to brucellosis infection in goats. Therefore, we propose that the identified SNPs within these genes could potentially influence their functions, and consequently, impact the animal’s response to the infection, particularly, in caprine brucellosis. This will deepen our understanding of regulation mechanisms and biological pathways involved in brucellosis infection in livestock.

It has been documented that genetic variations that influence cytokine production may be a useful aid in *Brucella* detection and protection [53]. The *SCLA111* gene displayed a higher level of mRNA in buffalos infected with brucellosis compared to those resistant to the disease [58]. Notably, the chronic form of bovine brucellosis was associated with increased expression of *IFN-γ*,* IL-1β*,* IL-6* and *iNOS* genes, along with reduced expression of *TNF-α*,* IL-4* and *IL-12p40* genes [63]. Furthermore, *Brucella* seropositive cows exhibited higher transcript abundances in *NRAMP1* and *iNOS* genes compared to seronegative cows [64]. This alteration of the regulation of inflammatory markers could be attributed to the host animal’s immune response to the *Brucella* infection. This immune response is initiated by the production of cytokines. Consequently, type-1 helper T (Th1) and natural killer (NK) cells express receptors for these cytokines and produce Interferon-γ (IFN-γ) in response to IL-12 or IL-23, which can be further enhanced by IL-1β and IL-18 [65]. IFN-γ, a versatile cytokine, plays a central role in type-1 immunity against intracellular pathogens like *Brucella* and upregulates macrophage-killing mechanisms by inducing the production of superoxide anions and hydrogen peroxide [66].

The changes observed in the mRNA levels of antioxidant markers could be attributed to the oxidative stress induced by *Brucella*. This stress contributes to tissue damage and increases the generation of free radicals and depletes antioxidants during the infection [67]. It has been documented that *Brucella* can stimulate the expression of *HMOX1* through the phosphoinositide 3-kinases/ glycogen synthase kinase-3 beta (PI3K/GSK3β,) AMP-activated protein kinase/ glycogen synthase kinase-3 beta (AMPK/GSK3β) and mitogen-activated protein kinase *(MAPK*) signaling pathways [68], which aids in its survival and growth. This may explain the observed alterations in *HO-1* gene expression in goats infected with *Brucella* in our study.

Erythritol has been associated with increased virulence in *Brucella* and was suggested as a potential factor contributing to the localization of *Brucella abortus* in the placenta of pregnant cows [69]. In the current study, we observed higher mRNA levels of the *TKT*, *RPIA* and *AMPD* gene in the infected does compared to healthy. Erythritol production was promoted by co-overexpressing of Ribose 5-Phosphate Isomerase A (*RPIA*) and transketolase (*TKT*) genes, both of which play a role in glycerol metabolism [70]. The AMP deaminase-encoding gene (*AMPD*) regulates the carbon flows in glycolysis and is crucial in the pentose phosphate pathway and erythritol synthesis [71]. Consequently, we harnessed the genetic resistance to brucellosis in goats by modulating the expression of the *TKT*, *RPIA* and *AMPD* genes.

The marked reduction in the levels of RBCs and hemoglobin concentration in *Brucella* infected does, accompanied by significant changes in HCT, MCV, MCH and MCHC is consistence with findings of other literatures [47, 72, 73]. This might be related to a decrease in the production of erythropoietin hormone, resulting in a reduced formation of RBCs. However, it is worth noting that a different outcome was observed in horse [74]. Conversely, a significant increase in the total leucocyte count in infected does may be attributed to the activation of the lymphoreticular system in response to the infection, which stimulates antibody production and cell-mediated immunity [73, 75]. Similar results have been reported in previous studies [47, 72]. additionally, the significant increasing in monocyte levels in *Brucella* infected does could be attributed to the presence of tissue debris in the uterus, with monocytes serving as scavengers [76]. These results are consistent with that obtained by [77, 78]. In contrast, there were significant decreases in TLC and neutrophils count, while there were significantly higher values of PCV, eosinophils and basophils in *Brucella*-infected cattle [79]. The values of Hb, PCV, TEC, TLC, lymphocytes and basophil reported in the current study fell within the range of reference values for *Brucella*-infected cattle [80].

The significant increase in serum activity of AST, ALT, GGT and ALP in *Brucella*-infected does in the current study may be attributed to liver damage caused by *Brucella*, resulting in an increase in these liver enzymes in the plasma [81]. Our results were inconsistent with those reported in previous study [47]. The activities of LDH showed a significant increase in infected does, possibly because of hemolysis, muscle damage and liver cell injury [82]. These findings are consistent with similar observations in cows [79, 83–86], goats [47, 87] and ewes [72, 88]. There was a significant reduction in serum levels of glucose, total proteins and albumin in *Brucella*-infected does, consists with previous findings in several studies [72, 85, 86, 88–90]. These reductions may be due to feed intake reduction in the infected does or impaired liver function [49].

The significant increase in serum globulin observed in the infected does could be explained by increasing the globulin fraction, particularly the γ-globulins [91], which is due to the bacterial infection as reported in previous studies [47, 86, 88, 89, 92]. Likewise, the high levels in serum triglycerides in brucellosis infected does in this study agree with previous studies [47, 72, 86, 87, 93]. This could be explained by the production of TNF-α [49, 94] in response to *Brucella* infection, which inhibits the lipoprotein lipase, then plasma triglyceride is increased [95]. The significant increasing concentration of serum cholesterol in brucellosis infected does consistent with previous studies [48, 87, 88, 90]. This could be attributed to cholesterol accumulation following hepatic damage caused by the infection. Moreover, hepatic tissue damage may also lead to a significant reduction in urea production, leading to decreases serum urea concentration in infected does [49]. These findings were similar to those documented in previous studies [19, 83, 86].

The significant decline in serum calcium and inorganic phosphorus levels observed in the infected does were consistence with those reported in previous studies [47, 85, 96, 97]. This could be due to changing of the pH in the small intestine, which impede the absorption of calcium and phosphorus [98]. In contrast, a significant reduction in serum sodium levels in the infected does while no significant changes were observed in potassium and magnesium levels. This decrease may be due to anorexia and fluid lost during abortion in the infected does [99]. Similarly, the present study reported significant decrease in the serum Fe, Cu and Zn in brucellosis infected does, which consistent with previous findings [73, 83, 85]. This may be due to liver dysfunction, disturbance of spleen function and a substantial bacterial uptake of Fe which is essential for the intracellular replication and virulence of *Brucella*. Additionally, the reduced values of serum Cu and Zn could be attributed to the chronic nature of the inflammatory process. Furthermore, the significant increase in NO level in the infected does compared to the non-infected was reported in previous studies [83, 100–103]. This may be because of the stimulation of NO synthesis in macrophage exposed to lipopolysaccharide [104].

The increase in MDA level in the current study may be attributed to the excessive production of free radicals during brucellosis, resulting in lipid peroxidation and formation of MDA [100]. Conversely, the reduction in TAC and GSH levels may be attributed to the oxidative stress that occurs during brucellosis leading to depletion of the antioxidant resources [105]. Furthermore, the reduction concentrations of Zn and Cu may be partially contributed to this process due to their essential role in antioxidants synthesis [103, 106, 107]. These results consistent with previous reports in cattle and sheep [47, 83, 101, 108]. The decrease in the enzymatic levels of SOD and GPx in *Brucella* infected does compared to non-infected could be due to the inhibition of certain cytokines [109] or the presence of type IV secretion system gene. This enhances the ability of the *Brucella* pathogen to invade and replicate within macrophages, thus, increases its virulence [110]. Similar observations were reported in camels [111] and sheep [103].

In this study, we selected the cytokines for analysis based on their pro-inflammatory (e.g., IL-1β and TNF-α) or anti-inflammatory (e.g., IL-10) elements. Notably, IL-1β and TNF-α showed a significant increase in *Brucella* infected compared to those who were not infected. In agreement, significant increases in TNF-α in the acute phase of brucellosis cases were reported [72, 101, 112, 113]. Conversely, the IL-10 level exhibited a significant decrease in *Brucella* infected compared to the non-infected does. This observation is consistent with previous studies [101, 114], which demonstrated that the persistent intracellular pathogen, *B. abortus*, prevents immune activation of the macrophage to produce IL-10 early in the infection. In vivo experiments also reported that the absent of endogenous IL-10 can boost the production of pro-inflammatory cytokines in mice [115] aiding in the clearance of *B. abortus*.

Haptoglobin and amyloid A serum values showed significant increase while fibrinogen was significantly decreased. These findings are consistent with previous results in sheep [103] and cattle [116]. Fibrinogen plays crucial role in various biological functions, including specific binding cites. It facilitates leukocyte binding to fibrinogen through integrin alpha and beta2, which have high partiality receptors on the monocytes and neutrophils [117]. In the current study, the significant increase in serum IL-6 level in the *Brucella* infected does refers to the significant role of these cytokines in the inflammatory response [118]. These results were consistent with previous reports [72]. Specific IgM antibodies typically develop early in the infection and persistent for several weeks to months [119], while IgG antibodies appear later and remain detectable for several months to years following the recovery [120]. In *Brucella* infected does, both IgM and IgG showed a significant increase compared to the non-infected ones. These results are in accordance with previous reports [19].

## Conclusion

The results of this study revealed significant differences in genes related to hematological, biochemical, immunological, and antioxidant functions between brucellosis-infected does with those that were not. Levels of *SLC11A1*,* TLR1*,* TLR9*,* SP110*,* ADORA3*,* CARD15*,* IRF3*,* HMOX1 TKT*,* RPIA and AMPD* were significantly upregulated in brucellosis-infected does compared to the non-infected. Conversely, *GPX1*, *NOS*, *NQO1* and *Nrf2* genes were significantly downregulated in brucellosis-infected does compared to the non-infected. Furthermore, this study also identified polymorphic variants in *SLC11A1*,* TLR1*,* TLR9*,* SP110*,* ADORA3*, *CARD15*, *IRF3*, *GPX1*, *NOS*, *NQO1*, *Nrf2*, *TKT*,* RPIA*, and *AMPD* genes that could be utilized to distinguish between does with and without *Brucella* infection. These findings introduced new genetic markers and putative candidate genes for identifying brucellosis infection in goats, suggesting that genetic variability between animals exists. These markers may be used as effective proxies for brucellosis in goats and open promising opportunities to control the disease through selective breeding programs. Importantly, further study with a larger sample size, multidisciplinary approaches and better identification of the causative agent is required to validate our results.

## Electronic supplementary material

Below is the link to the electronic supplementary material.

Supplementary Material 1: Table S1 Forward and reverse primer sequences, length of PCR product and annealing temperature for immune, antioxidant and erythritol related genes used in PCR-DNA sequencing. Table S2. Oligonucleotide primers sequence, accession number, annealing temperature and PCR product size of for immune, antioxidant and erythritol related genes used in real time PCR. Table S3. Distribution of SNPs, type of mutation in immune, antioxidant and erythritol related genes for tolerant and affected does to brucellosis

## Data Availability

Access to the data is available upon request from the corresponding author (ahmedsallam2@gmail.com).

## Abbreviations

RBC: Erythrocytes count
Hb: Hemoglobin
PCV: Packed cell volume
MCV: Mean corpuscular volume
MCH: Mean corpuscular hemoglobin
MCHC: Mean corpuscular hemoglobin concentration
WBC: Total leukocytes count
TP: Total protein
AST: Aspartate aminotransferase
ALT: Alanine transaminase
GGT: Gamma-glutamyl transferase
ALP: Alkaline phosphatase
LDH: Lactic dehydrogenase
Ca: Calcium
P: Phophprus
Mg: Magnesium
Na: Sodium
K: Potassium
Cu: Cupper
Zn: Zinc
Fe: Iron
GSH: Glutathione reduced
GPx: Glutathione peroxidase
MDA: Malondialdhyde
NO: Nitric oxide
SOD: Super oxide dismutase
TAC: Total antioxidant capacity
IL1-α: Interleukin 1 alpha
IL6: Interleukin 6
IL10: Interleukin 10
TNF-α: Tumor necrosis factor-alpha
IgG: Immunoglobulin G
IgM: Immunoglobulin M
Hp: Haptoglobin
SAA: Serum amyloid A
Fb: Fibrinogen
SLC11A1: Solute Carrier Family 11 Member 1
TLR1: Toll-like receptor 1
TLR9: Toll-like receptor 9
SP110: SP110 Nuclear Body Protein
ADORA3: The adenosine A3 receptor
CARD15: Caspase recruitment domain-containing protein 15
IRF3: Interferon regulatory factor 3
GPX1: Glutathione peroxidase 1
NOS: Nitric oxide synthetase
HMOX1: Heme Oxygenase-1
NQO1: NAD (P) H Quinone Dehydrogenase 1
Nrf2: Nuclear factor erythroid 2–related factor 2
TKT: Transketolase; RPIA = Ribose 5-Phosphate Isomerase A
AMPD: Adenosine monophosphate deaminase
CD14: Cluster of differentiation 14
CCL2: C-C motif ligand 2
SPP1: Secreted Phosphoprotein 1
BP1: Bactericidal permeability increasing protein
A2M: Alpha-2-Macroglobulin
ATP1A1: ATPase Na+/K + Transporting Subunit Alpha 1
TLR7: Toll-like receptor 7
TLR8: Toll-like receptor 8; β defensin = beta defensing
CCL2: Chemokine (C-C motif) ligand 2
SOD1: Superoxide dismutase 1
CAT: Catalase
AhpC/TSA: Alkyl hydroperoxide reductase/thiol-specifc antioxidant
PRDX2: Peroxiredoxin 2
PRDX4: Peroxiredoxin 4
NQO1: NAD (P) H Quinone Dehydrogenase 1
Nrf2: Nuclear factor erythroid 2–related factor 2
A: Alanine
C: Cisteine
D: Aspartic acid
E: Glutamic acid
F: Phenylalanine
G: Glycine
H: Histidine
I: Isoleucine
K: Lysine
L: Leucine
N: Asparagine
P: Proline
Q: Glutamine
R: Argnine
S: Serine
T: Threonine
V: Valine
