# Supplementary material for: Nucleotide sequence variants, gene expression and serum profile of immune and antioxidant markers associated with brucellosis resistance/susceptibility in Shami goat
Source: Ir Vet J. 2025 Jan 18;78:2. doi: 10.1186/s13620-025-00285-4 (PMC11748588; doi:10.1186/s13620-025-00285-4)
Supplement: Supplementary file 1 — Supplementary Material 1: Table S1 Forward and reverse primer sequences, length of PCR product and annealing temperature for immune, antioxidant and erythritol related genes used in PCR-DNA sequencing. Table S2. Oligonucleotide primers sequence, accession number, annealing temperature and PCR product size of for immune, antioxidant and erythritol related genes used in real time PCR. Table S3. Distribution of SNPs, type of mutation in immune, antioxidant and erythritol related genes for tolerant and affected does to brucellosis [file 13620_2025_285_MOESM1_ESM.docx]

**Supplementary tables**

**Table S1.** Forward and reverse primer sequences, length of PCR product and annealing temperature for immune, antioxidant and erythritol related genes used in PCR-DNA sequencing.

| Gene_ID | Forward Primer (5′-3′) | Reverse Primer (5′-3′) | AT (°C) | PCR product (bp) |
| --- | --- | --- | --- | --- |
| *SLC11A1* | GTGCCCGAGTCTGCAGTCCTCA | AGAGTGGGATTCGTCCGGCTGAG | 62 | 523 |
| *TLR1* | ATGCCTGACATCCTCTCACTATC | AGACAGTTCCAGACTCACTGTG | 60 | 471 |
| *TLR9* | ATGGGCCCCTACTGTGCCCCGC | TGCTGGTGCGGCTCAGCGACAG | 62 | 460 |
| *SP110* | TCCTGTTTTGCCCACTGGTATC | AGACACGGCAATAGCAGGGTC | 58 | 537 |
| *ADORA3* | CTACTCACCTGGGAAGCTTCTC | CCTTGACCTTCTCGTCCATGAC | 64 | 521 |
| *CARD15* | ATGTGCGCACAAGATGCTTTTCA | GCTGCTGTGTCAGGTCCAGCACA | 62 | 394 |
| *IRF3* | ACTCCTGGGAAGGATAAGCCCGA | AGGGCAGAAGACAGTCTGCTGGA | 60 | 468 |
| *GPX1* | CTCACTACTCTCAATTTGGAT | AGGCGAAGAGCGGATGCGCCTTC | 62 | 420 |
| *NOS* | GTGGAAGCGGTAACAAAGGAG | CTGCCATCTGGCATCTGGTAGCC | 64 | 332 |
| *HMOX1* | ATGGAGCGCCCGCAGCCCGACAG | TCACATTGCGTAAAGGCCCACAG | 60 | 867 |
| *NQO1* | GCAGCTCGCTAAGCAGCCTGAG | TACGAGCACCCGCTCAAACCAG | 62 | 466 |
| *Nrf2* | CTCAGTTACAACTAGATGAAGA | AATTATAACTGTCAATTTCTGTC | 60 | 480 |
| *TKT* | GCGCGGCTCCCGGGACTCTTAAC | GCAGGAAGCCAGCTTCAGCCCA | 60 | 414 |
| *RPIA* | ATGGGAACTTTATCCTGGACTG | AATAACAATACTTCAGAAGACTG | 60 | 338 |
| *AMPD* | GTCAAATTACAGGGGCAAAGCAG | TGTGGAACGGACAAACTAATAG | 58 | 382 |

- SLC11A1= Solute Carrier Family 11 Member 1; TLR1= Toll-like receptor 1; TLR9= Toll-like receptor 9; SP110= SP110 Nuclear Body Protein; ADORA3= The adenosine A3 receptor; CARD15= Caspase recruitment domain-containing protein 15; IRF3= Interferon regulatory factor 3; GPX1= Glutathione peroxidase 1; NOS= Nitric oxide synthetase; HMOX1= Heme Oxygenase-1; NQO1= NAD (P) H Quinone Dehydrogenase 1; Nrf2= nuclear factor erythroid 2–related factor 2; TKT= Transketolase; RPIA= Ribose 5-Phosphate Isomerase A; and AMPD= adenosine monophosphate deaminase.

AT=Annealing temperature

**Table S2.** Oligonucleotide primers sequence, accession number, annealing temperature and PCR product size of for immune, antioxidant and erythritol related genes used in real time PCR.

| **Gene** | **Primer** | **Product length (bp)** | **AT (°C)** | **Accession number** |
| --- | --- | --- | --- | --- |
| ***SLC11A1*** | F5′- GGACATCCGAGAAGCCAACA -3  R5′- TGGGAAAGATCGTCGCGTAG -3′ | 182 | 60 | [[KX151137.1](https://www.ncbi.nlm.nih.gov/entrez/viewer.fcgi?db=nucleotide&id=1070701429)](https://www.ncbi.nlm.nih.gov/nucleotide/KP682502.1?report=genbank&log$=nucltop&blast_rank=2&RID=GF4FXT0K014) |
| ***TLR1*** | F5′- ATGCCTGACATCCTCTCACT -3′  R5′- GAGGTTCAGAGTAGGGTGGC - 3′ | 162 | 58 | [NM_001285605.1](https://www.ncbi.nlm.nih.gov/entrez/viewer.fcgi?db=nucleotide&id=550822309) |
| ***TLR9*** | F5′- TTCACCTTGGACCTGTCACG -3′  R5′- ATTAACCGCCTGGGAGATGC - 3′ | 117 | 60 | [EU747825.1](https://www.ncbi.nlm.nih.gov/entrez/viewer.fcgi?db=nucleotide&id=190692982) |
| ***SP110*** | F5′- TGCCCACGATTCTTTCACGA -3′  R5′- CGGGCATAAATGAGCGAGGA - 3′ | 108 | 62 | XM_018058530.1 |
| ***ADORA3*** | F5′- TGAGGCTCTTCATTCTGCTCT-3′  R5′- CCAGTACTTGGGGTGGTCTT - 3′ | 143 | 58 | [XM_013962543.2](https://www.ncbi.nlm.nih.gov/entrez/viewer.fcgi?db=nucleotide&id=1062965089) |
| ***CARD15*** | F5′- TCCTGAGCACCTACGATGGA -3′  R5′- AAATGGTCACGGGTGCTGAA - 3′ | 157 | 60 | [NM_001314291.1](https://www.ncbi.nlm.nih.gov/entrez/viewer.fcgi?db=nucleotide&id=936975865) |
| ***IRF3*** | F5′- ACATGACTCCCGAGCAACTG -3′  R5′- TGGCAGCCTTCCACAATGAT - 3′ | 103 | 60 | JQ308793.1 |
| ***GPX1*** | F5′- AAGTTCATCACGTGGTCCCC-3′  R5′- CTGGGACAGCAGGGTTTCAA - 3′ | 153 | 58 | [XM_005695962.3](https://www.ncbi.nlm.nih.gov/entrez/viewer.fcgi?db=nucleotide&id=1062943833) |
| ***NOS*** | F5′- CGTTACGCCACCAACAATGG -3′  R5′- CCATCTGGCATCTGGTAGCC - 3′ | 131 | 60 | U29085.1 |
| ***HMOX1*** | F5′- TGAGCTGACCCGAGAAGGTT -3′  R5′- TAGAGGGGAGTGTAGACGGG - 3′ | 114 | 62 | [NM_001285567.1](https://www.ncbi.nlm.nih.gov/entrez/viewer.fcgi?db=nucleotide&id=550822278) |
| ***NQO1*** | F5′- TGCACTTCTGTGGCTTCCAA-3′  R5′- CCAGGCGTTTCTTCCATCCT - 3′ | 108 | 60 | [XM_005692193.3](https://www.ncbi.nlm.nih.gov/entrez/viewer.fcgi?db=nucleotide&id=1062927735) |
| ***Nrf2*** | F5′- CTACGGGCAAAAGCTCTCCA -3′  R5′- TCTGCAATTCTGAGCAGCCA - 3′ | 171 | 60 | KM576769.1 |
| ***TKT*** | F5′- GGCCGATCAGATCATCCAGG-3′  R5′- GCCTTGCGGGTAGCTATCTT - 3′ | 162 | 60 | [XM_018067049.1](https://www.ncbi.nlm.nih.gov/entrez/viewer.fcgi?db=nucleotide&id=550822069) |
| ***RPIA*** | F5′- GACGCTGACCTCAACCTCAT -3′  R5′- GTTCGGCTCACTGGGACATA - 3′ | 188 | 60 | XM_005686690.3 |
| ***AMPD*** | F5′- GTCCCTCCAGTGTCGTGTCT -3′  R5′- GCATTGCATCATCAATAGCTGGA - 3′ | 104 | 62 | XM_005677843.2 |
| ***ß. actin*** | F5′- CGTGCTGCTGACGGAGGCCCC-3′  R5′- GCACAGCCTGGATGGCCACATAC -3′ | 113 | 60 | AF481159.1 |

- - SLC11A1= Solute Carrier Family 11 Member 1; TLR1= Toll-like receptor 1; TLR9= Toll-like receptor 9; SP110= SP110 Nuclear Body Protein; ADORA3= The adenosine A3 receptor; CARD15= Caspase recruitment domain-containing protein 15; IRF3= Interferon regulatory factor 3; GPX1= Glutathione peroxidase 1; NOS= Nitric oxide synthetase; HMOX1= Heme Oxygenase-1; NQO1= NAD (P) H Quinone Dehydrogenase 1; Nrf2= nuclear factor erythroid 2–related factor 2; TKT= Transketolase; RPIA= Ribose 5-Phosphate Isomerase A; and AMPD= adenosine monophosphate deaminase.

**Table S3**. Distribution of SNPs, type of mutation in immune, antioxidant and erythritol related genes for tolerant and affected does to brucellosis.

| Gene | SNPs | Tolerant  N= 20 | Brucellosis  N=30 | Total | Type of mutation | Amino acid number and type |
| --- | --- | --- | --- | --- | --- | --- |
| *SLC11A1* | A27T | 12 | - | 12/50 | Synonymous | 27 S |
|  | C416T | - | 18 | 18/50 | Non-synonymous | 139 T to I |
| *TLR1* | A189T | - | 14 | 14/50 | Synonymous | 63 A |
|  | G285C | 8 | - | 8/50 | Non-synonymous | 95 Q to H |
|  | G308A | 17 |  | 17/50 | Non-synonymous | 103 S to N |
|  | G360C |  | 19 | 19/50 | Non-synonymous | 102 E to D |
|  | G394T | 15 |  | 15/50 | Non-synonymous | 132 V to F |
| *TLR9* | A174 G | - | 15 | 15/50 | Synonymous | 58 G |
| *SP110* | A62C | - | 24 | 24/50 | Non-synonymous | 21 N to T |
|  | C184T | - | 17 | 17/50 | Non-synonymous | 62 R to C |
| *ADORA3* | C387A | 16 | - | 16/50 | Synonymous | 129 I |
| *CARD15* | A73G | 11 | - | 11/50 | Non-synonymous | 25 S to G |
| *IRF3* | T133G | - | 21 | 21/50 | Non-synonymous | 45 F to V |
|  | C273G | 9 | - | 9/50 | Synonymous | 91 S |
| *GPX1* | G83A | 13 | - | 13/50 | Non-synonymous | 28 R to Q |
|  | T347C | - | 18 | 18/50 | Non-synonymous | 116 V to A |
| *NOS* | T63C | - | 13 | 13/50 | Synonymous | 21 L |
|  | T177C | 14 | - | 14/50 | Synonymous | 59 F |
| *NQO1* | A59C | 15 | - | 15/50 | Non-synonymous | 20 Q to P |
|  | G147A | - | 11 | 11/50 | Non-synonymous | 50 V to I |
|  | A380G | 8 | - | 8/50 | Non-synonymous | 127 N to S |
| *Nrf2* | C63G | - | 22 | 22/50 | Non-synonymous | 21 H to Q |
| *TKT* | C132T | 16 | - | 16/50 | Synonymous | 44 A |
|  | C215T | 12 | - | 12/50 | Non-synonymous | 72 P to L |
| *RPIA* | A195G | 10 | - | 10/50 | Non-synonymous | 65 T |
| *AMPD* | C224T | - | 19 | 19/50 | Non-synonymous | 75 A to V |

- CD14= cluster of differentiation 14; CCL2= C-C motif ligand 2; SPP1= Secreted Phosphoprotein 1; BP1= Bactericidal permeability increasing protein; A2M= Alpha-2-Macroglobulin; ATP1A1= ATPase Na+/K+ Transporting Subunit Alpha 1; TLR7= Toll-like receptor 7; TLR8= Toll-like receptor 8; β defensin= beta defensin; CCL2= Chemokine (C-C motif) ligand 2; SOD1= Superoxide dismutase 1; CAT; Catalase; AhpC/TSA—alkyl hydroperoxide reductase/thiol-specifc antioxidant; PRDX2= Peroxiredoxin 2; PRDX4= Peroxiredoxin 4; NQO1= NAD (P) H Quinone Dehydrogenase 1; and Nrf2= nuclear factor erythroid 2–related factor 2.

-A= Alanine; C= Cisteine; D= Aspartic acid; E= Glutamic acid; F= Phenylalanine; G= Glycine; H= Histidine; I= Isoleucine; K= Lysine; L= Leucine; N= Asparagine; P= Proline; Q= Glutamine; R= Argnine; R= Argnine; S= Serine; T= Threonine; and V= Valine.
